# Cardiovascular risk factors in fasting and postprandial lipid profiles among type 2 diabetes patients

**DOI:** 10.6026/973206300220922

**Published:** 2026-02-28

**Authors:** Sukh Dayal Kumhar, Praveen Kumar Tagore, Jitendra Kanjolia, Ashis Tiwari, Abha Bardiya, Shivani Parihar

**Affiliations:** 1 Department of General Medicine, Government Medical College, Datia, Madhya Pradesh, India; 2 Department of Anaesthesiology, Shyam Shah Medical College, Rewa, Madhya Pradesh, India; 3 Department of Pathology, Chirayu Medical College, Bhopal, Madhya Pradesh, India

**Keywords:** Type 2 diabetes mellitus, postprandial lipemia, cardiovascular risk, carotid intima-media thickness and triglycerides

## Abstract

Cardiovascular disease (CVD) dominates Type 2 Diabetes Mellitus (T2DM) mortality, yet fasting lipid profiles may miss atherogenic postprandial lipemia effects. Therefore, it is of interest to compare fasting versus postprandial lipids (4h post-high-fat meal) in 240 T2DM patients against Carotid Intima-Media Thickness (CIMT) as atherosclerosis surrogate. Fasting Low-Density lipoprotein (LDL) showed moderate CIMT correlation (r=0.68, p=0.001), but postprandial triglycerides/remnant cholesterol demonstrated stronger associations and independent multivariate prediction. Fasting triglycerides lacked significance. Postprandial lipids advance T2DM CVD risk assessment beyond fasting measures, supporting non-fasting protocols for better stratification.

## Background:

People with metabolic syndrome, also known as type 2 diabetes mellitus (T2DM), are two- to four-times more likely to suffer from cardiovascular disease (CVD) than those without the illness. One important factor that may be changed to reduce the risk in this group is dyslipidemia, which is characterized by the so-called diabetes lipid triad: high triglycerides (TG), low HDL-C, and small, dense lipoprotein (sdLDL) particles [1]. Conventionally, lipid management guidelines have centered on fasting lipid measurements with a particular emphasis on fasting LDL-C being the main risk stratification and treatment monitoring outcome [2]. Nevertheless, human beings spend most of their waking time in a postprandial (fed) condition. Metabolic flexibility is reduced in T2DM patients resulting in longer and magnified rises of glucose and lipid after meals [3]. There are indications that atherosclerosis is a postprandial effect. Postprandial hydrolysis of triglyceride-rich lipoproteins (TRLs) leads to the formation of remnant cholesterol particles, which become easily absorbed by the arterial intima in comparison with LDL and may have greater atherogenic potential because they are pro-inflammatory [4]. Recent epidemiological studies show that non-fasting triglycerides can be more useful predictors of risk of ischemic stroke and myocardial infarction as compared to fasting levels [5]. Nevertheless, the fasting measurements are mostly followed by the clinical practice, as the Friedewald equation of LDL calculation has been historically standardized and because of the uncertainty of variation in postprandial measurements [6]. According to a growing literature, dynamic alterations of lipids following a fat-load test depict the solubility ability of the metabolic system which in most cases is malfunctioning in insulin-resistant conditions [7]. Although the association between fasting lipids and the outcome of macrovascular has been well-documented, the strength of association between fasting and the postprandial lipid fractions and anatomical measurements of subclinical atherosclerosis, like Carotid Intima-Media Thickness (CIMT), has been poorly studied, especially in developing countries where the T2DM burden is increasing the most [8]. The proposed study will fill this research gap by conducting a comparative analysis of fasting and postprandial lipid levels of T2DM patients. Therefore, it is of interest to evaluate the statistical significance of the lipid state of fasting and postprandial with the CIMT, and thus assess the appropriateness of the two as cardiovascular risk factors.

## Materials and Methods:

A total of 240 patients with diagnosed Type 2 Diabetes Mellitus were enrolled. Study was conducted in Government medical College Datia M.P. India, start date of the research was September 2025, and end date was November 2025. The sample size was calculated based on a projected correlation coefficient of 0.25 between postprandial triglycerides and CIMT, with 80% power and a 5% significance level.

## Inclusion criteria:

[1] Adults aged 40 to 70 years.

[2] Diagnosis of T2DM for a minimum duration of 3 years.

[3] Patients on stable oral hypoglycemic agents (metformin, sulfonylureas, or DPP-4 inhibitors).

## Exclusion criteria:

[1] History of established cardiovascular events (myocardial infarction, stroke, or revascularization procedures).

[2] Severe renal impairment (eGFR < 30 mL/min/1.73m^2^) or nephrotic syndrome.

[3] Active liver disease or thyroid dysfunction.

[4] Patients currently on lipid-lowering therapy (statins or fibrates) were subjected to a 4-week washout period if deemed clinically safe; otherwise, they were excluded to prevent confounding of lipid metabolism.

[5] Pregnancy or lactation.

## Collection procedures of data:

## Anthropometry and baseline:

Demographic information, the length of diabetes, smoking history, and medication history were taken. Waist circumference and Body Mass Index (BMI) were measured in the WHO standards. Two measurements were taken and the average was taken as blood pressure.

## Lipid assessment protocol:

The participants were told to have 12 hours overnight fasting.

[1] Fasting Sample (0 hours): 8:00 AM Venous blood was sampled to measure fasting plasma glucose (FPG), HbA1c, and complete fasting lipid profile (Total Cholesterol, TG, HDL-C, and LDL-C).

[2] Standardized Meal Load: Within 20 minutes of the fasting draw, the patients were given a standardized high fat breakfast. The meal had about 800 kcal of energy with 50g of fat and 75g of carbohydrates and 25g of protein (made up of bread, butter, cheese and full-cream milk).

[3] Postprandial Sample (4 hours): A second venous blood sample was collected 4 hours after the meal, in order to measure postprandial lipid levels (PP-TG, PP-HDL, PP-Cholesterol).

## Laboratory analysis:

The enzymatic colorimetric analysis on an automated analyzer determined serum lipids. The Friedewald formula was used to obtain LDL-C levels when triglycerides were below 400mg/dl; direct LDL was obtained when triglycerides were above this value. The non-HDL cholesterol was computed as Total Cholesterol- HDL-C.

## Carotid intima-media thickness (CIMT):

An ultrasonography technique with a 7.5 MHz linear transducer was used by a single radiologist who was blinded to the results. One centimetre above the carotid bulb, on the far wall of the common carotid artery, was the CIMT recorded. To get the final CIMT value, three measurements were taken from the left and right carotid arteries and averaged. The value of CIMT, over 0.9 mm was believed to indicate a subclinical atherosclerosis.

## Statistical analysis:

The data was analyzed using SPSS version 25.0. Continuous variables were represented by means and standard deviations, whereas categorical variables were represented by percentages. Pairwise t-tests were used to compare fasting and postprandial lipid parameters. Use of the Pearson correlation coefficient (r) allowed us to ascertain the nature of the association between the lipid variables and CIMT. To identify the factors that might be used as independent predictors of CIMT, multiple linear regression analysis was performed. We considered the p-value to be statistically significant if it was less than 0.05.

## Results:

The sample population was 240 subjects with an average age of 56.4052 years. There was a minor male dominance (54.2%). The average types of diabetes were 7.8 years and the average HbA 1c was 7.9±1.2 which is moderation in controlling diabetes. The average weight was 28.1 kg/m^2^ which assigned most of the participants into the overweight group. The average CIMT of the cohort was 0.94 ±0.18 mm and 42 percent percentage of patients had CIMT greater than 0.9 mm. There was a remarkable change in the lipid parameters after standardized high-fat meal. The postprandial triglycerides (PP-TG) were much greater than the fasting triglycerides (F-TG), whose value increased between 158.4mg /dL and 245.6mg/dl (p<0.001). The postprandial state showed a statistically significant but numerically negligible drop in HDL-C levels, which is consistent with this. In comparison to fasting levels, postprandial total cholesterol and non-HDL cholesterol were significantly higher (Table 1). According to the results of the Pearson correlation analysis, the relationship between CIMT and the lipid markers in the postprandial state was greater than in the fasting condition. While there was a modest positive association between Fasting LDL-C and CIMT (r=0.38), Postprandial Triglycerides showed a much stronger positive correlation (r=0.65, p<0.001), which was much higher than the Fasting Triglycerides correlation (r=0.41). Postprandial non-HDL cholesterol was similarly strongly correlated (r=0.59) (Table 2). Age, Body Mass Index (BMI), Hemoglobin A1c (HbA1c), Systolic Blood Pressure (SBP), Fasting LDL (FL), and Postprandial Triglycerides (PGT) were the independent variables that were included in a multiple linear regression model that was developed to find CIMT predictors. Postprandial triglycerides were shown to be a stronger independent predictor of CIMT (0.42, p=0.001) than fasting low-density lipoprotein (0.21, p=0.03), according to the research. When the fasting triglycerides were inserted into the model, they lost significance when other risk factors were considered (Table 3).

## Discussion:

It has shown that the current research demonstrates that postprandial lipids, especially triglycerides, are better predictors of subclinical atherosclerosis in Type 2 Diabetes Mellitus patients than the conventional fasting variables. The high increase in triglycerides and non-HDL cholesterol four hours following a high-fat meal indicates a failure of the clearance of triglyceride-rich lipoproteins (TRLs) which has been sometimes referred to as postprandial dysmetabolism [9]. The fact that Postprandial TG has a stronger correlation with CIMT (r=0.65) than Fasting TG (r=0.41) is in line with current knowledge about atherogenesis. Fasting TG is a static parameter that is an estimate of the hepatic secretion of VLDL, and postprandial concentrations represent the dynamism between intestinal chylomicron secretion and remnant particle clearance [10]. The insulin resistance in T2DM reduces the activity of lipoprotein lipase (LPL), which results in chylomicron lipid remnants and VLDL lipid remnants. These remnant particles are even smaller than nascent chylomicron and can enter the endothelial barrier, being retained in the sub endothelial space, triggering macrophage uptake and the foam cell formation without prior oxidation, as do native LDL [11]. Strong correlations between CIMT and postprandial non-HDL cholesterol were also noted. Apo lipoprotein B (apoB)-carrying atherogenic lipoproteins, including LDL, VLDL, IDL and lipoprotein (a), are collectively known as non-HDL cholesterol. In our regression analysis, we observed that postprandial non-HDL had a higher predictive power than fasting LDL. This supports the recommendations made by the European Atherosclerosis Society (EAS), which contends that non-fasting samples are sufficient, and potentially even superior, for predicting cardiovascular risks [12]. Interestingly, we discovered a little decrease in HDL-C in the postprandial situation. This reduction is likely due to the fact that cholesteryl ester transfer protein (CETP) mediates the exchange of triglycerides in TRLs for cholesteryl esters in HDL. The effect of this process is the development of triglyceride-enriched HDL that are quickly broken down by hepatic lipase to smaller and less protective HDL particle and resultant into elevated HDL-C blood concentrations, a factor that further increases the risk of cardiovascular disease [13].

We have found that the Fasting LDL is not the sole target to be used to treat diabetic patients. Although LDL is the main cause of plaque formation, the remaining cardiovascular risk in patients treated with statin is usually thought to be caused by non-LDL factors, namely TRL remnants [14]. Clinicians can fail to appreciate the cumulative number of atherogenic particles that a patient is subjected to in a day by overlooking the postprandial condition. The Nordestgaard studies have been known to provide evidence that non-fasting triglycerides are causal factors of ischemic heart disease and all-cause mortality [15]. These epidemiological relationships in a diabetic cohort have structural validation in our anatomical data by CIMT. Weaknesses of this study are that it was a cross-sectional study, and thus it does not allow one to establish causality. Although CIMT is a proven surrogate of generalized atherosclerosis, it is never accurate in predicting the occurrence of plaque rupture. Also, the standardized meal, which will be used in this research, might not represent the varied food habits of the general population but can be compared under control. Longitudinal research is required in future to establish whether the specific reduction of hard cardiovascular endpoints (MI/Stroke) by targeting postprandial lipids occurs in T2DM [16, 17- 18].

## Conclusion:

Postprandial lipid levels, in particular triglycerides and non-HDL cholesterol levels, are more accurate predictors of carotid intima- media thickness than fasting lipid levels in Type 2 Diabetes Mellitus. The defects within the lipoprotein metabolism that cannot be detected during fasting are revealed in the postprandial state. As a result, there is a possibility of underestimating cardiovascular risk in diabetic patients due to the exclusive use of fasting lipid profiles.

## Figures and Tables

**Table 1:** Comparison of fasting and postprandial lipid parameters (n=240)

**Parameter (mg/dL)**	**Fasting (Mean ± SD)**	**Postprandial (4h) (Mean ± SD)**	**Mean Difference**	**P-value**
Total Cholesterol	185.2 ± 35.4	196.8 ± 38.1	11.6	<0.001
Triglycerides	158.4 ± 52.6	245.6 ± 78.9	87.2	<0.001
HDL Cholesterol	42.5 ± 9.1	39.8 ± 8.5	-2.7	0.002
LDL Cholesterol	111.0 ± 28.3	108.5 ± 29.1	-2.5	0.15
Non-HDL Cholesterol	142.7 ± 32.5	157.0 ± 36.4	14.3	<0.001
Note: LDL was calculated;
direct measurement verified
in high TG samples.

**Table 2:** Pearson correlation coefficients (r) of lipid parameters with CIMT

**Lipid Parameter**	**Fasting State (r)**	**P-value**	**Postprandial State (r)**	**P-value**
Total Cholesterol	0.32	<0.01	0.39	<0.01
Triglycerides	0.41	<0.001	0.65	<0.001
HDL Cholesterol	-0.28	0.02	-0.34	<0.01
LDL Cholesterol	0.38	<0.001	0.35	<0.01
Non-HDL Cholesterol	0.44	<0.001	0.59	<0.001

**Table 3:** Multiple linear regression analysis for predictors of CIMT

**Variable**	**Standardized Coefficient (β)**	**t-value**	**P-value**	**95% CI**
Age	0.28	4.12	<0.001	0.005 - 0.014
HbA1c	0.15	2.35	0.02	0.010 - 0.052
Systolic BP	0.18	2.88	<0.01	0.001 - 0.004
Fasting LDL-C	0.21	2.15	0.03	0.001 - 0.003
Postprandial TG	0.42	5.67	<0.001	0.002 - 0.005
BMI	0.09	1.45	0.14	-0.002 - 0.012
